# Table Manners

**Published:** 1891-10

**Authors:** 


### TABLE MANNERS.

A great many children are not properly trained in table manners; and it must always be the fault of the mother—if there be one—that this is so. Let no one plead that she has not the time for such instruction; a mother must pay the price of maternity, and has no moral right to shirk her obligations, nor to let other matters crowd them aside. There is a right and a wrong way to do every thing. The wrong way to go about this is to wait till the child has become fixed in awkward and slovenly habits, and then try to correct them by sharp reprimands or punishment. One cannot begin too soon after the child begins to eat to teach it to take its food daintily; to hold the spoon and fork correctly, using the latter whenever possible; to eat slowly, avoiding all unnecessary noises, movements, or exhibitions of the act of mastication. Like unto these rules, and quite as important, are those points of deportment which do not really include the partaking of food. To wait quietly until helped; not to clamor for desired articles, or to tease for them when refused; not to overstock the plate, or desire more than can be eaten; to talk little unless spoken to, especially at a strange table; never to leave the table before the others, unless asking and obtaining permission. These well known essentials are necessary among civilized beings, if one is to enjoy both the companionship and the food at meals. A mother who has the right iufluence over the little one can so instruct and prepare him when there are no observers. It is apt to embarrass, if not "harden" a child, to have the attention of company directed toward it by a reproof. Mistakes or misconduct should be noticed and pointed out in private. A few months of constant vigilance is a small price to pay for correct habits of eating, and will save both mother and child a great deal of regret in after life.

